# Role of Matrix Metalloproteinase 9 in Predicting Lymph Node Metastases in Oral Squamous Cell Carcinoma

**DOI:** 10.7759/cureus.33495

**Published:** 2023-01-07

**Authors:** Satadruti Chakraborty, Turuvekere Narayan Rao Suresh, Azeem S Mohiyuddin

**Affiliations:** 1 Pathology, Sri Devaraj Urs Medical College, Kolar, IND; 2 Otolaryngology and Head and Neck Surgery, Sri Devaraj Urs Medical College, Kolar, IND

**Keywords:** biomarker, tnm staging, lymph node metastasis, mmp9, oral cancer

## Abstract

Background

Oral cancer is a common malignancy worldwide, with approximately 3,50,000 new cases diagnosed yearly. Out of many factors which affect the survival in patients with oral cancer, lymph node metastases are a major factor that reduces survival by 50%. Even though many biomarkers have been studied to predict lymph node metastasis, none have yet been accepted for routine use. Matrix metalloproteinases (MMPs) play a vital role in extracellular matrix (ECM) degradation, thus facilitating the invasive potential and the metastatic cascade of tumors. Of the different subtypes, multiple studies have demonstrated that matrix metalloproteinase 9 (MMP9) overexpression is often associated with the aggressive nature of the tumor. Therefore, this investigation is done to know the role of MMP9 in predicting lymph node metastasis in oral squamous cell carcinoma (OSCC).

Aim

To determine the immunohistochemical expression of MMP9 in OSCC and to find its association with lymph node metastasis.

Settings and design

It is a laboratory-based observational study.

Materials and methods

One hundred five histologically proven cases of OSCC were studied. Histopathological parameters like depth of invasion, presence of lymph node metastasis, grading, and TNM staging were done according to the 8th AJCC staging criteria. Both intensity and proportion of MMP9 expression were recorded.

Statistical analysis

For qualitative data, the Chi-square test was used as a test of significance. The p-value (probability that the result is true) of <0.05 was considered statistically significant after assuming all the rules of statistical tests.

Results

A higher expression of MMP9 was observed in 56.2% of cases and the higher expression correlated with the presence of lymph node metastases (p<0.001), an advanced stage of cancer (P <0.001), and grade of the tumor (p=0.003).

Conclusion

A positive association between MMP9 and lymph node metastasis and pathological TNM staging demonstrates MMP9 as a potential biomarker to predict the behavior of the tumor.

## Introduction

Oral squamous cell carcinoma (OSCC) is one of the most prevalent cancers in the world, annually affecting 300,000 people with approximately 150,000 deaths [1]. Out of many factors like late diagnosis and local recurrence that influence patient survival in oral carcinoma patients, arguably lymph node metastasis can be considered a major prognostic determinant, affecting the survival rate by reducing it by 50% [2].

Cancer metastases require cancer cells to break down extracellular matrix (ECM) structures. During cancer cell invasion and metastasis, tumor cells digest the ECM molecules by synthesizing and employing various enzymes. The matrix metalloproteinases (MMPs) are the main contributors to ECM degradation by tumor cells [3]. Matrix metalloproteinase 9 (MMP9) is a 92 kDa gelatinase that plays important roles in tumor invasion and angiogenesis. It is linked to the aggressiveness of many cancers. It leads to type IV collagen degradation, a principal component of basement membranes [4-6]. Monoclonal antibodies developed against MMP9 have been studied in many cancers, of which moderate efficacy has been observed in gastric carcinoma when given along with cytotoxic drugs. Similar developments in OSCC can warrant an improvement in patient outcomes and prognosis. Therefore, a study of the expression of MMP9 in OSCC can help predict the patient's prognosis and plan treatment.

## Materials and methods

Methodology

This study was conducted in the Department of Pathology. It included 105 cases of resected specimens with modified radical neck dissection (MRND), fixed in 10% neutral buffered formalin, which have been histopathologically diagnosed as OSCC. All the patients were clinically staged between T2 and T4b. The H&E slides from the primary tumor were screened for the selection of representative blocks for immunohistochemistry (IHC) staining with MMP9. IHC staining was performed on 10% formalin-fixed, paraffin-embedded 4-micrometer tissue sections. Tissue sections after deparaffinization in xylene were rehydrated through a descending ethanol series (100, 95, 90, 80, and 70%) at room temperature for five minutes. Antigen retrieval was done using a microwave, followed by a wash in distilled water after allowing it to cool for 10 minutes. Endogenous peroxidase activity was blocked with hydrogen peroxide for 5 minutes. Incubation with primary rabbit prediluted monoclonal (Clone: EP127, PathnSitu Biotechnologies, CA, USA) MMP9 antibody for 30 minutes at room temperature was done. The slides were then rinsed with Tris-buffered saline (TBS) three times and then incubated with secondary antibody reagent: conjugated rabbit and anti-mouse polymer horseradish peroxidase (HRP) secondary antibody for 15-30 minutes at room temperature. DAB was applied for minutes. For counterstaining, the slides were then rinsed with deionized water. Incubated for 5 minutes with Hematoxylin and rinsed with TBS buffer for 1 minute. 

Grading of IHC

For the interpretation of the immunoreactivity, five representative fields from the tumor were chosen for evaluating intensity (I) and distribution (D) of cytoplasmic staining of the tumor cells by two independent pathologists, and a semi-quantitative scoring was performed for both the intensity and the proportion of staining of the tumor cells ranging 0-3. In case of disparity in the interpretation of IHC, a consensus was reached between the two pathologists. 

The intensity of the cytoplasmic staining of the tumor cells was graded as 0 = None, 1 = Mild, 2 = Moderate, and 3 = High.

The percentage of tumor cells showing cytoplasmic staining was graded as follows: 0 = None, 1 = <10%, 2 = 10-50%, 3 = >50%.

The final staining scores were assigned based on the multiplication of the staining intensity and the percentage of the positive cells and graded as follows: 0 :0; 1: 1-3; 2: 5-6; 3: 7-9. A total expression score of 0 or 1 was considered a low expression and a score of 2 or 3 was considered as high expression [1].

Statistical analysis

After entering data into Microsoft Excel, data analysis was done with SPSS 22 version software. Frequencies and proportions were used to represent categorical data. For qualitative data, the Chi-square test and Fisher's exact test (for 2x2 tables only) were used as a test of significance. P-value (probability that the result is true) of <0.05 was considered statistically significant after assuming all the rules of statistical tests. Statistical software: MS Excel, SPSS version 22 (IBM SPSS Statistics, Somers, NY, USA) was used to analyze data.

## Results

Most patients were in their sixth decade of life (64%), with a female predominance, having a male:female ratio of 1:4. The most common site encountered was buccal mucosa (54%).

**Figure 1:**
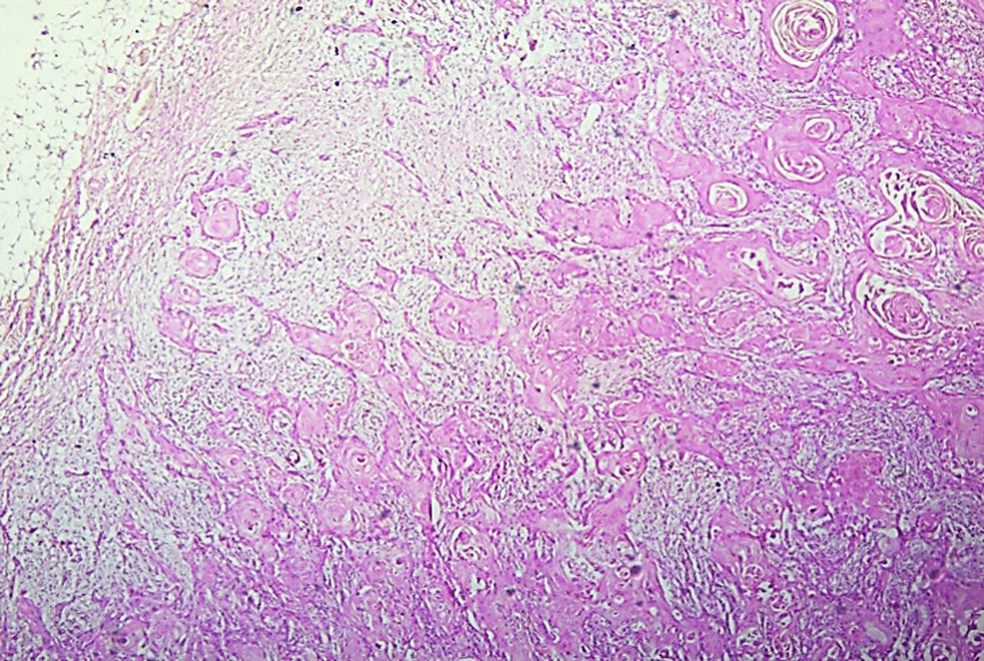
Squamous cell carcinoma deposits in lymph node (H&E 100x).

Out of the total 105 cases, the majority, i.e., 81, was diagnosed as well-differentiated SCC (77%), 23 as moderately differentiated (21%), and only one as poorly differentiated SCC. Forty-eight of the total 105 cases with a frequency percentage of 45.7% had lymph node metastasis (Figure 1), with nine of them showing extra-nodal extension.

The microscopic depth of invasion was divided into three groups in which 25 cases had less than 5 mm of the depth of invasion, 51 cases had less than 6-10 mm of the depth of invasion, and 27 cases had more than 10 mm.

**Figure 2:**
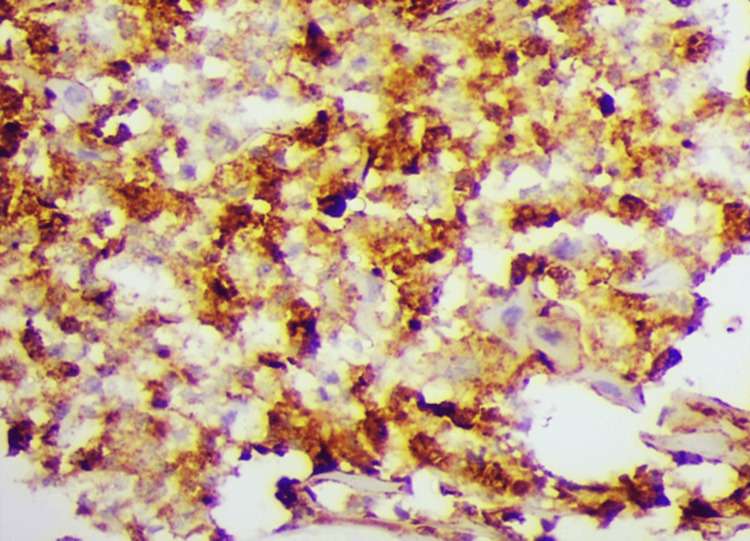
Tumor cells showing a high expression of MMP9 (IHC 400x). IHC: Immunohistochemistry; MMP9: Matrix metalloproteinase 9.

The 8th AJCC criteria were used for final TNM staging, and most cases, i.e., 33 (31.4%), were stage II and stage IV, closely followed by (28.5%) in stage III. Expression of MMP9 protein was observed in 102 cases. However, a high expression was observed in 59 cases (56.1%) (Figure 2).

With respect to the presence of lymph node metastasis, among the 59 patients who had a high expression of the marker, 45 (76.3%) had lymph node metastases, and among the 46 patients who had a low expression, 43 (93.4%) were found to have no lymph node metastases. This showed a statistically significant association (p<0.001) between a high expression of the marker and the presence of lymph node metastases (Table 1). Similarly, a higher expression of the marker was observed in TNM stages III and IV with a statistically significant association (p<0.001) between both parameters (Table 1). With respect to the grade of the tumor, the expression of the marker was increased in moderately and poorly differentiated tumors while decreased in well-differentiated tumors, establishing a statistically significant association (p=0.03) between higher expression of the marker and the grading of the tumor (Table 1).

**Table 1:** Expression of MMP9 with respect to the presence of lymph node metastasis, tumor grade, and TNM staging. MMP9: Matrix metalloproteinase 9.

Parameter		Low Expression	High Expression	Total	P-value
Lymph node	N_0_	43 (93.4%)	14 (23.7%)	57	p <0.001
	N_+_	3 (6.6%)	45 (76.3%)	48	
	Total:	46	59	105	
Grade	Well Differentiated	41 (89.1%)	40 (67.7%)	81	p = 0.003
	Moderately Differentiated	05 (10.9%)	18 (30.5%)	23	
	Poorly Differentiated	00	01 (1.8%)	01	
	Total	46	59	105	
TNM Stage	Stage I	7 (15.2%)	03 (5%)	10	p <0.001
	Stage II	24 (52.1%)	09 (15.2%)	33	
	Stage III	11 (23.9%)	18 (30.5%)	29	
	Stage IV	4 (8.8%)	29 (49.3%)	33	
	Total:	46	59	105	

## Discussion

MMPs are a group of zinc endopeptidases vital for the breakdown of ECM components and basement membrane. Studies have demonstrated that MMP9 is a key prognostic factor for various cancers such as retinoblastoma, bladder cancer, and ovarian epithelial cancer [7-9]. In a meta-analysis done by Wei Deng and his colleagues on overexpression of MMPs in Oral cancer patients, have included 15 studies evaluating different types of MMPs concerning 1266 patients. They concluded that MMP 9 overexpression correlated with poor prognosis in Oral cancer patients and thus suggested that evaluation of the marker in the patients can help to predict the prognosis [10].

Studies conducted on expression of MMP 9 in OSCC showed a higher expression ranging from 50% to 75% of the cases and low expression ranging from 25% to 40% of the cases. while the expression of MMP9 in this study was evaluated in a total of 105 cases, majority of them i.e., 59 (56.2%) showed a high expression and rest 46 (43.8%) cases showed a low expression [11-13]

In this study, 76.3% of the cases showing a high expression of CD44 had lymph node metastases, and 93.4% of cases showing a low expression of CD44 had no lymph node metastases. Similarly, in the study by Ravi DK et al., among the total 100 cases, 88.23% of the cases with lymph node metastases had a high expression, and 11.77% had a low expression [11]. The total number of cases without lymph node metastases was 15, all of which had a low marker expression.

In the study conducted by Atla B et al. [14], 70% of the cases with lymph node metastases had a high expression of the marker, and the remaining 30% had a low expression. Among the cases without lymph node metastases, 82.2% had a low expression, and 17.8% had a high expression of the marker.

In another study conducted by Sowmya V et al. [15], among the 20 cases with lymph node metastases, 85% showed a high expression, and 15% showed a low expression of the marker. The 20 cases with N0 Nodal status showed a high expression in 30%, and 70% had a low expression of the marker (Table 2).

**Table 2:** Correlation of MMP9 expression with lymph node metastases

Name of the author	Total no. of cases	High Expression			Low Expression			P value
Ravi Dk et al^11^	100	85			15			P < 0.001
		N_+_:75		N_0_:10	N_+_:0	N_0_:15		
Atla B et al^14^	65	20			45			P <0.05
		N_+_:14		N_0_:6	N_+_:8		N_0_:37	
Sowmya V et al^15^	40	20			20			P < 0.001
		N_+_:14		N_0_:6	N_+_:6		N_0_:14	
Present study	105	59			46			P <0.001
		N_+_:45	N_0_:14		N_+_:3		N_0_:43	

In this study, the higher TNM stages correlated with higher expression of MMP9. A total of 52.1% with low expression belonged to stage II, and 49.3% with high expression belonged to stage IV. This established a statistically significant association between a high expression of the marker and the advanced stage of the tumor (p<0.001). These findings are in accordance with studies conducted by Atla B et al. [14] and Sowmya V et al. [15], which showed a significant association between the expression of the protein and an advanced stage of the tumor.

The initial step in a metastatic process involves dislodging the tumor cells from the primary tumor site, followed by improvement in their invasive potential [16]. The invasion process is facilitated by the degradation of the pericellular and extracellular matrix. This degradation is mainly brought about by MMP9, along with other proteolytic enzymes [17]. MMP9 also plays a role in the intravasation of the cancer cells to the blood vessels and lymphatics by helping the cancer cells degrade the vessel wall, following which the cancer cells adhere themselves to the vessel wall and then, with the help of MMP9 and highly proteolytic MT1-MMP, the tumor cells extravasate out of the vessels and migrate to the distant sites [18-19]. In addition to these functions, MMP9 is also known to promote the formation of new lymphatic vessels, which potentiates further metastases of the tumor cells [20].

With such evidence demonstrating the role of MMP9 in metastases, synthetic inhibitors of the protein are being developed and implemented in clinical trials over the past 30 years. The first generation of these drugs had an inhibitory effect on all the members of MMPs, thus impacting the normal physiological processes [21]. Further research yielded the development of drugs specifically against MMP9, and the effect of humanized monoclonal antibodies (ANDECALIXIMAB) against the enzyme has been studied in gastric carcinoma, which showed moderate efficacy when they were given in combination with other cytotoxic drugs [22]. Similarly, the establishment of targeted therapy against the protein can play a major role in improving the mortality and morbidity among OSCC patients.

## Conclusions

Cervical lymph node metastasis is an important factor contributing to morbidity and mortality in OSCC patients. As observed in this study, a high expression of the MMP9 is associated with a higher chance of lymph node metastases and an advanced tumor stage. Thus, expression of the MMP9 on biopsy may help the oncosurgeon with treatment planning.
